# Empathy, Challenge, and Psychophysiological Activation in Therapist–Client Interaction

**DOI:** 10.3389/fpsyg.2018.00530

**Published:** 2018-04-11

**Authors:** Liisa Voutilainen, Pentti Henttonen, Mikko Kahri, Niklas Ravaja, Mikko Sams, Anssi Peräkylä

**Affiliations:** ^1^Faculty of Social Sciences, University of Helsinki, Helsinki, Finland; ^2^Faculty of Arts, University of Helsinki, Helsinki, Finland; ^3^Department of Psychology and Logopedics, Faculty of Medicine, University of Helsinki, Helsinki, Finland; ^4^Department of Neuroscience and Biomedical Engineering, Aalto University School of Science, Espoo, Finland

**Keywords:** empathy, challenge, psychotherapy, psychophysiology, social interaction, autonomic nervous system activation

## Abstract

Two central dimensions in psychotherapeutic work are a therapist’s empathy with clients and challenging their judgments. We investigated how they influence psychophysiological responses in the participants. Data were from psychodynamic therapy sessions, 24 sessions from 5 dyads, from which 694 therapist’s interventions were coded. Heart rate and electrodermal activity (EDA) of the participants were used to index emotional arousal. Facial muscle activity (electromyography) was used to index positive and negative emotional facial expressions. Electrophysiological data were analyzed in two time frames: (a) during the therapists’ interventions and (b) across the whole psychotherapy session. Both empathy and challenge had an effect on psychophysiological responses in the participants. Therapists’ empathy decreased clients’ and increased their own EDA across the session. Therapists’ challenge increased their own EDA in response to the interventions, but not across the sessions. Clients, on the other hand, did not respond to challenges during interventions, but challenges tended to increase EDA across a session. Furthermore, there was an interaction effect between empathy and challenge. Heart rate decreased and positive facial expressions increased in sessions where empathy and challenge were coupled, i.e., the amount of both empathy and challenge was either high or low. This suggests that these two variables work together. The results highlight the therapeutic functions and interrelation of empathy and challenge, and in line with the dyadic system theory by Beebe and Lachmann (2002), the systemic linkage between interactional expression and individual regulation of emotion.

## Introduction

Psychotherapy is done through social interaction. While the form and content of therapists’ responses to their clients’ experience may vary greatly, among other possible distinctions, psychotherapist’s attitudes toward the client can be divided to two basic orientations or facets: empathy and challenge (Bänninger-Huber and Widmer, 1999; Voutilainen et al., 2010; Ribeiro et al., 2013; Weiste and Peräkylä, 2014). To empathize means that the therapist attunes him- or herself to the client’s experience; to challenge means that the therapist, sometimes overtly but often discreetly, questions the client’s beliefs about self, the world and his or her ways of being with others. Different clinical theories offer conceptualizations of this dichotomy (e.g., Greenson, 1967; Beck, 1976; Warner, 1997; Greenberg, 2004; Stern, 2004). Despite the difference in conceptualizations, both empathy and challenge are observable in the actual interaction across different psychotherapy approaches (Bänninger-Huber and Widmer, 1999; Voutilainen et al., 2010; Ribeiro et al., 2013; Weiste and Peräkylä, 2014) and both have positive association with therapeutic outcome (Orlinsky and Howard, 1986; Keijsers et al., 2000; Elliot et al., 2011). Although empathy and challenge can be seen as diverging orientations, in reality they often co-exist (Bänninger-Huber and Widmer, 1999; Voutilainen et al., 2010; Ribeiro et al., 2016).

We presume that the therapist’s empathetic and challenging behaviors are linked to the mental and bodily emotion systems of the participants. This paper will focus on that linkage. We ask how empathy and challenge in the therapists’ interventions connect to emotion-related physiological responses (indicating valence and arousal) in the participants during these interventions, and more globally during the therapy sessions.

The dyadic systems theory by Beebe and Lachmann (2002) proposes that interactional regulation of emotion and the participants’ self-regulation of emotion are systemically linked. According to this theory, means of regulating expressions of emotion in the interaction are, simultaneously, means of regulating inner emotional experiences and physiological arousal. This hypothesis has been supported in recent empirical studies from non-clinical interactions that showed correlations between storytelling interaction and psychophysiological responses in the participants, suggesting that people share the “emotional load” of storytelling through story recipients’ behavioral and bodily responses to the emotion that the storyteller conveys in the story (Voutilainen et al., 2014; Peräkylä et al., 2015). Focusing on the co-regulation of emotion in psychotherapy, we enhance understanding of the clinical process: the ways in which therapists help the clients to regulate and work with their emotions, and the ways in which client’s emotion system reacts to interaction. Our study is also relevant for understanding the therapists’ physiological-emotional work load.

The structure of the paper is as follows: we will next discuss earlier research on psychophysiology and social interaction in psychotherapy, present our micro-analytical approach [conversation analysis (CA)], and hypotheses. In the method section we discuss the data collection, interaction variables, and the method of the analysis. We will then present our results from two time frames: (1) across the sessions and (2) during interventions. Finally, we will discuss the results with regard to earlier research.

### Emotion, Psychophysiology, and Social Interaction

In accordance with the prevalent psychological view, we break down the variety of emotion into two key dimensions: *valence* and *arousal* (Larsen and Diener, 1992; Barrett and Russel, 2009). Valence refers to the hedonic tone of the emotion varying from pleasant (positive) or to unpleasant (negative). Arousal refers to the felt activation associated with an emotional experience, varying from low to high. The valence and arousal dimensions of emotion are anchored in different organizations of the human body. The activation of the facial muscles in the brow, cheek, and periocular regions is linked to the valence (smile and frown) (Bradley and Lang, 2007, pp. 590–592). The arousal component, then, involves the activation of the autonomic nervous system, detectible for example in heart rate, electrodermal activity (EDA, often known as skin conductance), frequency of breathing, and perspiration (Bradley and Lang, 2007, pp. 587–590).

In psychophysiological research on psychotherapy, some studies focus on reactivity in clients: Steffen et al. (2014) showed that clients display increased physiological reactivity to stress relative to a control group; on the other hand, psychotherapy is effective in decreasing stress reactivity (Aubert-Khalfa et al., 2008; Cyranowski et al., 2009). Even more important for the study at hand is the long research tradition of clients’ and therapists’ psychophysiological reactions during actual psychotherapy sessions (for overviews, see Cacioppo et al., 1991; Kleinbub, 2017). In 1950’s scholars in the United States started to investigate how participants’ talk in psychotherapy sessions was linked to their physiological reactions (Boyd and DiMascio, 1954; DiMascio et al., 1955, 1957; Coleman et al., 1956; for an early review, see Lacey, 1959). For example DiMascio et al. (1957) showed that during sessions where the client more frequently ‘shows tension,’ heart rate of both the client and the therapist tended to be higher than during sessions with less frequent client displays of tension. ‘Showing antagonism’ in the client’s talk made it likely that the heart rate of the client went up, while the heart rate of the therapist went down.

In a study of 43 sessions of a psychotherapy client, Dittes (1957) found that the therapist’s *permissiveness and friendliness* during the therapy hour decreased the client’s EDA. Furthermore, in a study of initial interviews with 12 clients with their 12 therapists and a series of psychotherapeutic interviews with one client, McCarron and Appel (1971) showed that the therapist’s verbalizations evoke different EDA responses in the client, which are in accordance with a high to low magnitude hierarchy of *confrontation*, *interpretation*, *interrogation*, and *reflection*. The results also indicated a similar correspondence between the therapist’s own autonomic arousal and the hierarchy of the verbalizations. Regarding the clients’ activation, the pattern was further elaborated in an experimental study simulating clinical interaction (Olson and Claiborn, 1990). The subjects responded by physiological arousal to confrontations. When an interpretation was given after a confrontation, the subjects’ level of arousal decreased. More recently, Villmann et al. (2008) linked *emotional intensity* in interactions to increased variation in psychophysiological responses, and Päivinen et al. (2016) linked *blaming the partner* in couple therapy to increased electrodermal activation in the clients and the therapists.

Physiological synchrony between the client and the therapist is another important area of research (for a systematic review, see Kleinbub, 2017). Marci et al. (2007) showed a correlation between therapist empathy, as it was perceived by the client, and increased synchrony in skin conductance changes of the therapist and the client. Likewise, Messina et al. (2012) found that (pseudo-) patients’ perceived empathy correlated with synchrony in skin conductance in simulated clinical sessions. Furthermore, Messina et al. (2012) compared EDA synchrony in simulated sessions with a therapist, a psychologist, and a non-therapist, showing that while psychologists showed higher levels of synchrony with 0-s lag, psychotherapists showed higher levels of synchrony with 3-s lag. The authors suggest that this reflects more effortful, controlled reflection in the empathetic responses by the psychotherapists. Karvonen et al. (2015) studied synchrony in EDA in a couple-therapy context where the therapy was conducted by two co-therapists. The authors compared the synchrony in EDA between different dyads formed from the four participants. Against the authors’ expectation, synchrony was highest between the two co-therapists and lowest between the spouses. The authors consider it possible that the therapists’ shared focus of attention on the clients might account for their high synchrony (see also Seikkula et al., 2015).

Linkages between empathy and psychophysiology have been studied also in contexts other than therapy. Finset et al. (2011) examined empathy and psychophysiological activation in clinical interviews, comparing empathetic vs. neutral style. Contrary to what was expected, the study showed an increase in EDA in the patients in the empathy condition. The authors interpreted that this reflected heightened positive emotion in the empathetic interviews. In a yet different setting, Peräkylä et al. (2015) investigated psychophysiological activation during conversational storytelling in dyads where the participants were asked to talk freely about their life events. Recipients of the stories shared the “emotional load” of the storytelling, as the recipients’ affiliation with the storytellers’ emotion, shown in overt verbal and non-verbal behavior, led to a decrease in the storytellers’ and increase in recipients’ EDA levels.

Importantly, the time frame of the analysis varies. Most existing studies operate with the whole encounters (be they psychotherapy sessions or other interactions). Some take a different direction, focusing on shorter events within larger encounters: participants’ psychophysiological responses during interventions (McCarron and Appel, 1971; Olson and Claiborn, 1990), during narratives (Peräkylä et al., 2015), or particular topical units in the session (Seikkula et al., 2015).

As whole, the picture provided by earlier research is not quite unified. For example, empathy has been variably connected to an increase and decrease in arousal, and in a divergence in arousal in the participants. In a recent systematic review of the literature, Kleinbub (2017) wrote that the study of interpersonal physiology in psychotherapy lacks consensus “in almost every aspect, expect the existence of the phenomenon.” Most salient finding in the study of interpersonal autonomic physiology seems to be the link between empathy and psychophysiological synchrony in psychotherapy and in other contexts (Kleinbub, 2017; Palumbo et al., 2017).

In the current study, we will investigate the interplay of empathy, challenge, and psychophysiological responses in therapists and clients, leaving aside the complex questions of synchrony. We draw our hypotheses from earlier research that suggests that empathy and challenge connect to dyadic internal regulation of emotion. More specifically, as in Peräkylä et al. (2015) on storytelling, we expect empathy to increase arousal of the participant giving empathy (i.e., the therapist) and to decrease it in the participant receiving empathy (i.e., the patient). Likewise, drawing from the earlier studies showing that confrontation (and other actions that go to unexpected directions) is associated with increased arousal, we expect that the therapists’ challenging actions will increase physiological arousal in both parties. As for the timescale of the analysis, we will analyze the participants’ responses both across the whole session, and focusing on a shorter time span around particular events – therapist interventions – during the sessions. This will give us a possibility to consider the immediate physiological reflections of the therapist’s interactional moves (physiological activation during different actions), as well as their slower physiological effects (how the “dose” of different actions shows in the physiological activation in individuals at the session-level).

Besides autonomic arousal, we will analyze facial muscle activation (EMG) associated with the valence of emotional expression (smile and frown). Earlier experimental research using EMG has linked increase in frown activation to empathy (Sun et al., 2015) and mental effort (Van Boxtel and Jessurun, 1993). In a quasi-experimental study on conversational storytelling in dyads, story recipients’ facial activation (smile and frown) was found to reflect the valence of the storyteller’s affective stance (Voutilainen et al., 2014). In the current study, we expect that empathy increases the therapists’ facial activation and clients’ positive emotion, whereas challenge increases frown and decreases smile activations in both participants.

In his recent review, (Kleinbub, 2017) suggested that the psychophysiological study of psychotherapy might benefit from increased attention to micro-processes in therapy interaction, including “data-driven procedures of content classification” (Kleinbub, 2017). While we don’t follow Kleinbub’s (2017) suggestion to focus on differences between individual patients, the study at hand involves an effort to an enhanced focus on micro-processes and data-driven procedures. We are interested in empathy and challenge as they appear in the therapist’s interventions, but in contrast to the earlier studies, we consider empathy and challenge as qualities that can characterize wide variety of interventions. In the identification of these interventions we rely on CA that is a particularly context-sensitive approach to study interaction (Sidnell, 2010; Peräkylä, 2012).

### Formulating Client’s Talk

Our micro-analytical approach on psychotherapy interaction is based on CA. CA is a social scientific and linguistic method for the study of the practices and processes of social interaction (see Sidnell, 2010). It gives us the means to analyze in detail the timing and design of different kinds of therapeutic actions and their interactional consequences, using video and audio recordings of therapy sessions as data (Peräkylä, 2012; Voutilainen and Peräkylä, 2014).

Even though empathy and challenge can be conveyed at any moment and by any means, including facial expressions (Bänninger-Huber and Widmer, 1999) and prosody (Weiste and Peräkylä, 2014), they become particularly salient in therapists’ verbal interventions. Here we will limit our focus on frequent interventions that in CA are called *formulations* (see Antaki, 2008). According to Heritage and Watson (1979) formulations are utterances in which the current speaker suggests a meaning of what another participant has said in the prior turn or turns, and this is something psychotherapists quite often do. A formulation is inevitably selective: it foregrounds something in the prior talk, and leaves something else in the background. In the current study, we use the term formulation in a broad sense, referring to turns with different syntactic structures (besides declaratives, they may be interrogative) in which the therapists display what they have heard the client saying in their earlier talk. Furthermore, we have not made a sharp distinction between formulations and *extensions* (therapist’s utterances that as it were continue the client’s turn at talk, see Vehviläinen, 2003; Peräkylä, 2008) and *interpretations* (in which the therapist communicates his or her own perspective toward what the client has expressed, see Vehviläinen et al., 2008, cf. Stiles, 1992), as extensions and interpretations often also include formulation of the patient’s prior talk. Therefore, for the current paper, any therapist utterance where he or she shows his or her understanding of the clients talk is a formulation, even if it also would do the work that extensions and interpretations do.

Previous CA research on psychotherapy has shown that formulations often convey empathetic understanding of the client’s experience, and/or challenge the client (Vehviläinen, 2003; Peräkylä, 2011; Weiste and Peräkylä, 2013, 2014; cf. Ribeiro et al., 2013; Muntigl et al., 2014). It is important to notice, however, that to empathize and to challenge *are not mutually exclusive functions* (see, e.g., Ruusuvuori, 2007; Voutilainen et al., 2010; Peräkylä, 2011). In this paper, we therefore consider empathy and challenge as two independent dimensions of formulations that can also co-occur. We investigate the relation of these dimensions to the physiological activation in the participants.

### Extending the Research Field

While the study of psychophysiological processes in psychotherapy has a long history, the results of the studies thus far remain scattered (Kleinbub, 2017). We seek to expand the research tradition in three ways. (1) By introducing a sensitive micro-analytic method (CA) for the analysis of the relevant interactional events in the psychotherapy sessions. Thereby, we aim at tracing the relevant interactional events on a new level of precision. (2) By including two time frames in our study, including both immediate (singular) effects of empathy and challenge, and their cumulative effects during full sessions. Thereby, we seek to achieve a fuller picture of the physiological responses patterns in interaction. (3) By investigating empathy and challenge in the same interactional events (therapists’ interventions), and not treating them as different actions. Thereby, our analysis can be more valid clinically, as in the actual clinical work empathy and challenge are often mixed.

### Hypotheses

Based on the earlier research reviewed above, we expected that (1) during the interventions, and (2) during the whole therapy sessions

(1) Empathy, viewed from the formulations by observers, decreases the clients’ and increases the therapists’ physiological arousal (as indexed by EDA and heart rate).
(2) Empathy increases positive emotional facial expressions (as indexed by orbicularis oculi muscle activity) and decreases negative emotional expressions (as indexed by corrugator supercilii muscle activity) in both participants.
(3) Challenge, viewed from the formulations by CA coders, increases physiological arousal (EDA and heart rate) in both participants.
(4) Challenge decreases positive emotional expressions (orbicularis oculi muscle activity) and increases negative emotional expressions (corrugator supercilii muscle activity) in both participants.

## Materials and Methods

### Participants

The data were recorded from psychodynamic psychotherapy sessions. The participants were five dyads. The therapists were experienced private psychotherapists in their fifties and sixties with advanced level psychotherapy training (acknowledged by the Finnish National Supervisory Authority for Welfare and Health) and long experience in psychodynamic psychotherapy. They all had post-graduate degrees (from medicine or psychology) alongside their psychotherapy training. The participants were sitting during the sessions, the frequency of which was once or twice a week. Two clients suffered primarily from depression, one from anxiety disorder, one from eating disorder and one from obsessive-compulsive disorder. Four of the clients were in their twenties, one in her sixties. The data include one dyad of a male therapist and a male client, two dyads of a female therapist and a female client and two dyads of a male therapist and a female client.

Written informed consent was obtained from all participants in accordance with the Declaration of Helsinki. They reviewed oral and written information about the research, and they were given the opportunity to cancel the consent for any reason. When the data was collected, there was no relevant ethics committee in Finland for this study, because the study was done at a social science department and did not involve patients in public health care (all therapies were in private practice). However, another study (Stevanovic et al., unpublished) that was part of the same project used identical procedure of data collection, data management and publication practices. This study involved patients in public health care, and was thus under the auspices of the Ethical Board of Helsinki University Hospital, which gave its permission to the study on 21.09.2011. From each dyad six subsequent sessions were recorded. The sessions were from different phases of the therapies: one from the early phase, three from the middle and one in the ending phase of the therapy. From these 30 sessions, six sessions were used in a preliminary data-driven analysis of the interaction, building of coding schemes, and in the training of coders. This training set was excluded from the final coding and analysis of the data. For building the coding scheme, we selected first two sessions from one of the dyads and then the first session from the other dyads. The rest of the sessions (24) were used in the actual coding and analysis.

### Psychophysiological Data Collection

Data were recorded with two Nexus-10 amplifiers (Mind Media BV, Netherlands). EDA signal was recorded at 32 Hz with two electrodes affixed to middle phalanges of the non-dominant hand. ECG signal was recorded at 512 Hz using a modified Lead II electrode placement. Facial EMG data were recorded at 2048 Hz with bipolar electrodes placed at *orbicularis oculi* and *corrugator supercilii* muscle locations on the left side of the face. Physiological data were synchronized with the audiovisual recording using digital markers from the computer running the video collection.

Data preprocessing was conducted with Matlab 8.5. The EDA data were smoothed with a low-pass filter at 1 Hz. ECG signal was filtered and R-peaks were detected using the ECGLAB (De Carvalho et al., 2002) toolbox. EMG signals were low-pass filtered at 20 Hz using 3rd order Butterworth filter and rectified. The quality of all signals was manually inspected by an experienced researcher. Short movement artifacts in EDA were interpolated and ectopic and misdetected beats in ECG were removed from the analysis.

### Conversation Analysis

Conversation analysis is mostly a qualitative research method, but it can be applied also in quantitative research, as in the current study. The method is context-sensitive in terms of the actual interaction, but independent from any theories of psychotherapy. The basic principle in CA is to observe the relation between adjacent turns at talk; the social actions are not identified as separated from their context, but in relation to what has been said before, most importantly in the just preceding turn. This was done in this study too. This kind of context-sensitivity to the previous utterance sets challenges to the reliability of coding the interaction, but on the other hand it leaves freedom for the coders to interpret the situation from the perspective of the participants (see Stivers, 2015). We find this especially important in the study of such subtle phenomenon as challenge in psychotherapy.

### Interactional Measures

#### Formulations

Applying CA, we coded all interventions in which the therapists communicate to the client what they have heard the client said in his/her previous talk. The formulations can have declarative or interrogative forms but their primary function is not to elicit information but to offer a rephrased version of the client’s talk. Coders of the data (Voutilainen and Kahri) are conversation analysts with 5–10 years’ experience of the method. The coders did not have clinical background, and the coding scheme was independent from clinical reasoning.

#### Challenge

Challenge was coded from all formulations. We assessed whether a formulation is challenging or not. On the basis of qualitative analysis of the data, we built a coding scheme that identified three basic ways in which the psychodynamic therapists challenge the clients’ preceding talk: (1) therapist can formulate the problematic emotion as more intense than expressed by the client, (2) therapist can shift the focus from an external referent to an internal referent (experience) of the client, or (3) therapist can formulate the content of the clients’ experience as different than the client had described it as. If the therapist’s formulation performed one or more of these it was coded as challenging. The challenge can be very discreet and overlap with empathetic understanding of the client. Obviously, interpretation by the coders was needed. This interpretation, however, was based on CA (what is observable from details of interaction, e.g., word choice and main referents in utterances), not clinical reasoning. Coders (Voutilainen and Kahri) were the same as in coding of formulations. First the coders coded sessions of the training set (excluded from the analysis) and discussed cases of disagreement before coding independently the rest of the sessions.

From the 24 coded sessions, 694 formulations were coded. Of the formulations, 455 were coded as non-challenging (“benign”) and 239 as challenging. Ten of the 24 sessions were double coded. Cohen’s (1968) κ statistic was computed to assess the nominal inter-coder reliability. The mean κ was 0.47 (0.23–0.84) indexing “fair to good” reliability (75% hit rate). To our understanding, the apparently common disagreement between the coders reflects the inherent vagueness of the challenge that was coded; we find a lot of borderline cases between “benign” formulations that just rephrase the client’s words and formulations that change the content, referent or emotional intensity of the client’s talk. Despite this vagueness, however, we wanted to maintain coding that allows interpretation from the coders and thus context-sensitivity that is crucial in CA.

#### Empathy

Unlike the identification of formulations and challenge, the measurement of empathy was not based on CA coding but on the judgment of naïve raters. We considered rating, rather than coding, as apt for measuring empathy, because of the multimodality of empathetic displays (e.g., Peräkylä and Sorjonen, 2012) and understanding that ordinary persons are competent for recognizing emotional events. Raters were asked to assess on the scale of 1–9 how empathetic the therapist appeared in each coded formulation. The instruction was modified from the instruction for raters used in an earlier study by Peräkylä et al. (2015). The raters were instructed to rely on their first impression in their evaluation. Empathy was paraphrased as “the therapist as it were takes part in the client’s emotions.” Two raters (university students) saw a short segment of the preceding talk and focus interventions in a random order from all five therapists. ICC (3,k) for inter-rater reliability was 0.58, indicating fair reliability, close to the limit of good reliability in 0.60 (Cicchetti, 1994). We considered this sufficient level of agreement. Given that the rating focused on short segments of interaction the context of which was not shown to the rater, higher level of agreement might be hard to attain. Furthermore, the psychodynamic approach of the therapists entailed certain minimalism of emotional expression, so the differences that the raters were attending to were discreet.

### Statistical Analysis

For the analysis of physiological responses to the whole session (tonic responses), we calculated the mean skin conductance level (SCL), heart rate [beats per minute (bpm)], and root mean square (RMS) value of EMG activity for each session. In addition, we quantified changes in arousal (as indexed by SCL and heart rate) across the session. The EDA signal was downsampled (by factor of 8) to 4 Hz and cardiac (inter-beat-interval) signal was interpolated to form a 4-Hz uniformly sampled time series and converted to heart rate (bpm). Coefficient of the least squares regression line (i.e., linear trend) during session, multiplied by time and thus resulting in difference between the start and end point values in original units, was used as an index of change across a session. Logarithmic transformations were conducted for the data to normalize the distributions where appropriate. The interactional variables were the ratio of challenging formulations for a session and mean empathy of the formulations during a session.

When analyzing physiological responses to interventions (phasic responses), as the length of the interventions differed, we focused on the last part of an intervention where the content of the intervention is apparent and the client begins to respond to the possible challenge. We calculated the mean physiological values for 10-s epochs ranging from 5 s before to 5 s after the end of an intervention. The interactional variables were the type of formulation (challenging/“benign”) and empathy score of the formulation.

Physiological data were analyzed by the linear mixed models (LMM) procedure in SPSS with restricted maximum likelihood estimation. When analyzing physiological responses to the whole session, role nested within dyad was specified as the subject variable and session number was specified as the repeated variable, and AR(1) was specified (on the basis of Schwarz’s Bayesian Criterion, BIC) as the covariance structure for the residuals. When analyzing physiological responses to interventions, role nested within dyad and session were specified as the subject variables and intervention number was specified as the repeated variable, and ARMA(1,1) was specified as the covariance structure for the residuals. Role (therapist, patient), challenge (the ratio of challenging formulations; or challenging, non-challenging formulation), therapist empathy (rating), and Role × Challenge, Role × Empathy, and Challenge × Empathy interactions were specified as fixed effects.

## Results

The data supported many of our hypotheses but also provided unexpected results. Most importantly, the change in the participants’ physiological activation across a session was as we expected: the therapist’s empathy increased the therapist’s physiological activation and decreased the client’s physiological activation. We also found that the therapist’s arousal increased during the challenging interventions. Furthermore, there was an interaction effect of empathy and challenge, suggesting that these variables work together. Besides the statistically significant results, we also report trends in the data, considering also them of importance given the small database and the natural (non-experimental) setting of the research.

### Change in Activation Across a Session

#### Skin Conductance

**FIGURE 1:**
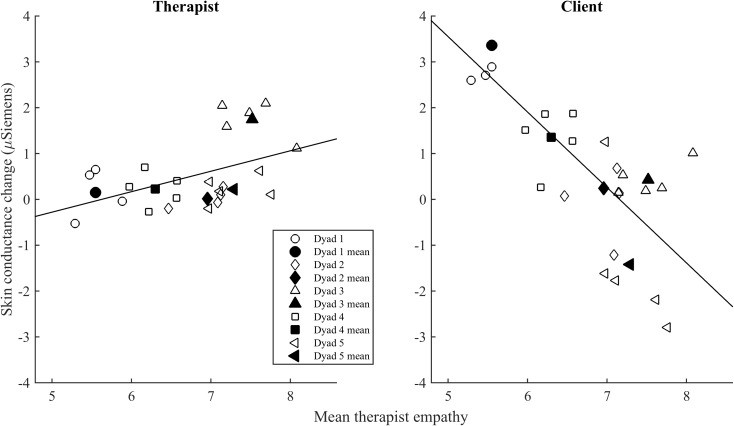
The relationship of mean empathy with change in skin conductance level (SCL) across a session. The open symbols indicate the mean of empathy and SCL change in each session for the therapist and the client. The filled symbols indicate the mean empathy and SCL change of the therapist and the client in each dyad.

Our hypothesis on empathy and physiological arousal was confirmed regarding the change in SCL across a session. Role of the participant [*F*(1,13.46) = 14.68, *p* < 0.01] and Role × Empathy [*F*(1,12.61) = 15.57, *p* < 0.01] significantly predicted SCL change. Larger mean therapist empathy led to an increase in SC levels in therapists, whereas in patients it led to decrease of EDA. **Figure 1** illustrates the effect of empathy on the change in SCL across a session for therapists and clients.

**FIGURE 2:**
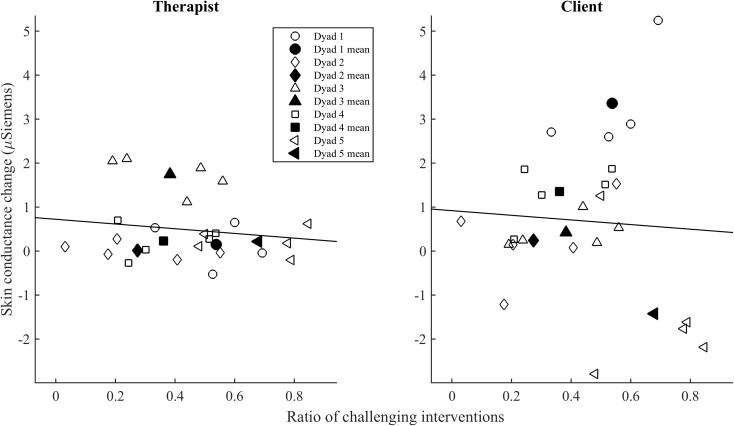
The relationship of ratio of challenging interventions with change in SCL across a session. The symbols indicate the mean of challenge ratio and mean SCL in each session for the therapist and the client. The colored symbols indicate the mean of the therapist and the client in each dyad.

Regarding our hypothesis on challenge, a higher ratio of challenging interventions tended to be associated with an increase in SCL across a session, but this effect failed to reach statistical significance [*F*(1,38.60) = 3.09, *p* < 0.10; see **Figure 2**]. This appeared to be the case primarily for the clients (four of the five dyads as shows in the figure 2 below) rather than the therapists.

In addition, a higher ratio of challenging interventions tended to lead to a larger increase in SCL across the session especially when empathy was low, but the interaction between challenge and empathy failed to reach statistical significance [*F*(1,38.69) = 3.31, *p* < 0.10]. There was no significant effect of empathy or challenge on the mean SCL across a session (overall activation).

**FIGURE 3:**
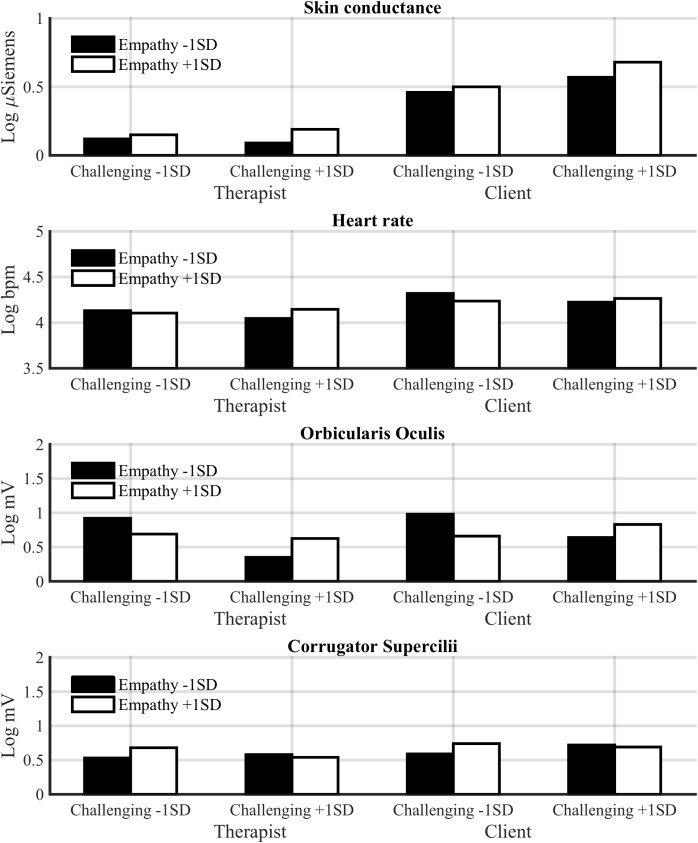
Estimated mean physiological values for low-challenge (M –1 SD) and high-challenge (M +1 SD) sessions as a function of empathy for the therapist and client.

**FIGURE 4:**
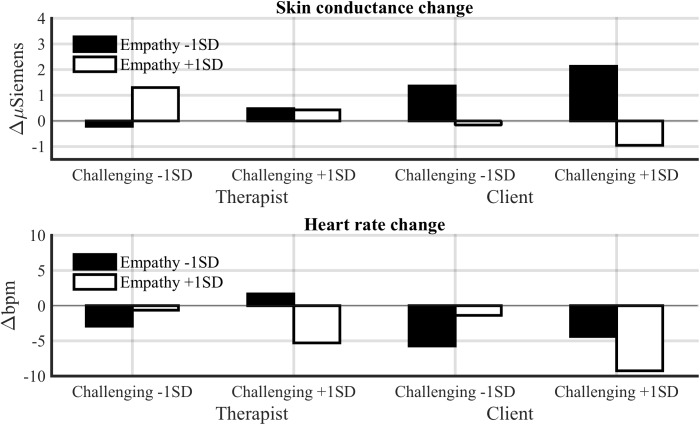
Estimated change in SCL and heart period across a session for low-challenge (M –1 SD) and high-challenge (M +1 SD) sessions as a function of empathy for the therapist and client.

**Figure 3** shows mean physiological values and **Figure 4** changes in SCL and heart rate across the session for low-challenge and high-challenge sessions as a function of empathy.

#### Heart Rate

As we hypothesized, challenge increased heart rate of the participants. Challenge was associated with both an increase in heart rate across a session [*F*(1,27.24) = 8.52, *p* < 0.01] and high mean heart rate [*F*(1,30.10) = 5.27, *p* < 0.05]. Also empathy increased heart rate of the participants across a session [*F*(1,23.65) = 5.82, *p* < 0.05]; thus, the hypothesis that empathy would decrease the client’s heart rate was not supported. Unexpectedly, empathy and challenge also interacted. In sessions with low empathy and a low ratio of challenging interventions, and sessions with high empathy and a high ratio of challenging interventions, the heart rate of the participants decreased across the sessions [*F*(1,26.91) = 9.96, *p* < 0.01]. On the other hand, in these sessions the mean heart rate of both participants was higher [*F*(1,29.80) = 5.26, *p* < 0.05]. This suggests that empathy and challenge are connected also at the physiological level. We will return to this in the discussion.

#### Orbicularis Oculi EMG

As hypothesized, empathy was positively associated with orbicularis oculi EMG activity across sessions [*F*(1,34.77) = 8.24, *p* < 0.01], whereas challenge was negatively associated with it [*F*(1,27.12) = 15.33, *p* < 0.001]. Also the Challenge × Empathy interaction was significant [*F*(1,27.04) = 13.49, *p* < 0.001]. As was the case for heart rate, empathy was related to high orbicularis oculi activity across sessions with a high ratio of challenging responses, but the reverse was true for sessions with low challenge.

#### Corrugator Supercilii EMG

Although challenge tended to be associated with higher corrugator EMG activity across sessions, the association was non-significant [*F*(1,29.80) = 2.76, *p* = 0.11]. Also empathy tended to be positively associated corrugator activity, but the effect was non-significant [*F*(1,32.04) = 2.67, *p* = 0.11].

### Responses to Interventions

#### Skin Conductance

**FIGURE 5:**
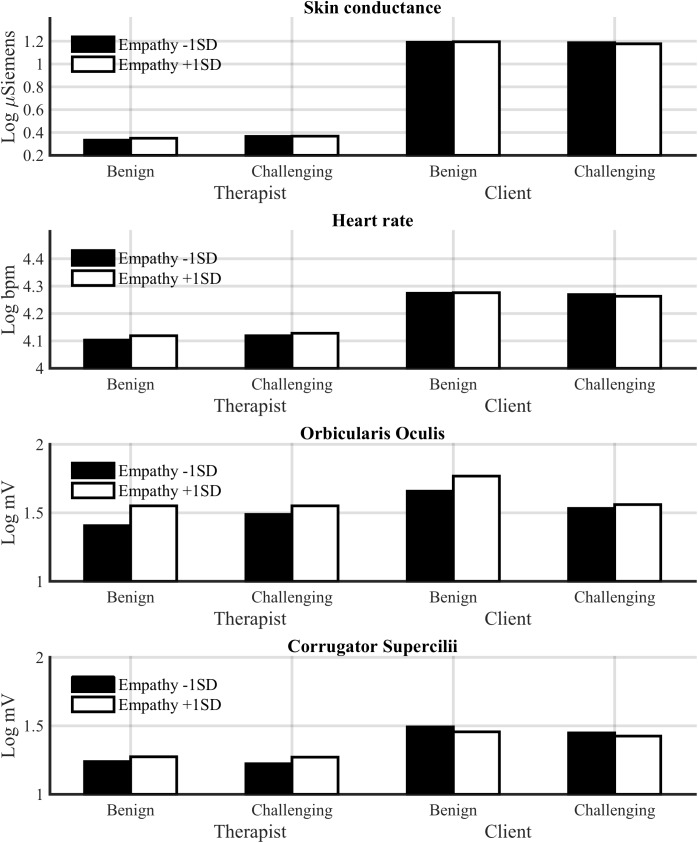
Estimated physiological responses to benign and challenging interventions as a function of empathy for the therapist and client.

Therapists had a significantly lower SCL (*M* = 0.36, SE = 0.09) in response to all interventions compared to clients [*M* = 1.19, SE = 0.09, *F*(1,48.21) = 45.01, *p* < 0.001]. Our hypothesis that challenge would increase SCL in both participants was partly supported. Challenge and role interacted significantly [*F*(1,1224.64) = 11.69, *p* < 0.001; see **Figure 5**]. A *post hoc* comparison showed that challenging interventions increased SCL in therapists (*p* < 0.001), but not in clients. Empathy was not related to SCL elicited by interventions.

#### Heart Rate

Therapists had a significantly lower heart rate (*M* = 4.12, SE = 0.03) compared to clients [*M* = 4.27, SE = 0.03, *F*(1,53.57) = 15.89, *p* < 0.001]. The positive association of empathy with heart rate failed to reach statistical significance [*F*(1,1494.82) = 2.78, *p* < 0.10]. However, the Empathy × Role interaction was significant [*F*(1,1495.81) = 6.10, *p* < 0.01], indicating that empathy increased heart rate of therapists, but not of clients. The main effect of challenge was a non-significant trend [*F*(1,1500.88) = 2.74, *p* < 0.10], but the Challenge × Role interaction was significant [*F*(1,1525.33) = 15.21, *p* < 0.001]. Challenging interventions increased heart rate in therapists (*p* < 0.001) and decreased it in clients (*p* < 0.05). Thus, both empathy and challenge seem to increase the therapist’s, but not client’s, physiological arousal in response to interventions (formulations).

#### Orbicularis Oculi EMG

Empathy was positively associated with orbicularis oculi EMG responses (positive emotional expressions) to interventions [*F*(1,1520.99) = 9.86, *p* < 0.001]. Empathy tended to increase orbicularis oculi activity more in response to non-challenging interventions, but the Challenge × Empathy interaction failed to reach significance [*F*(1,1495.30) = 2.93, *p* < 0.10]. The interaction of challenge and role was significant [*F*(1,1559.66) = 15.94, *p* < 0.001]; challenging interventions decreased orbicularis oculi activity only in clients. Thus, although empathy and challenge did not influence a client’s arousal during interventions, they appear to influence the client’s positive emotional expressions.

#### Corrugator Supercilii EMG

Corrugator Supercilii activity indexing negative emotional expressions tended to lower in the therapists compared to clients, although the difference was not significant [*F*(1,76.77) = 3.46, *p* < 0.10]. Also, empathy tended to increase corrugator activity in therapists, whereas the reverse tended to be the case in clients, although the Empathy × Role interaction failed to reach significance [*F*(1,1553.07) = 2.71, *p* = 0.10].

**Figure 5** shows these results on responses to interventions.

## Discussion

The results confirmed our hypotheses in four respects: (a) *empathy* increased the therapist’s and decreased the client’s skin conductance over the session, (b) empathy increased orbicularis oculi EMG activity indexing positive emotional expressions (and possibly a positive internal emotional state) in both the therapists and the client across the sessions, (c) *challenge* increased heart rate of both the therapist and client across the sessions, and (d) challenge increased the therapist’s skin conductance during the very intervention. Although the hypotheses were not confirmed in all respects, the results support our general hypothesis that the therapist’s empathy is connected to positive valence and decrease in arousal in the client, and that the therapist’s challenge is connected to increase in physiological arousal in the participants. This is in line with how clinical literature describes the effect of these two basic orientations of therapeutic work (Bänninger-Huber and Widmer, 1999; Ribeiro et al., 2013). Our study offers clear evidence that therapists and clients orient to empathy and challenge not only in the verbal level of interaction, but also physiologically.

In particular, the result on the effect of empathy at the session level suggests that the phenomenon of “sharing the emotional load” that has been found from everyday storytelling (Peräkylä et al., 2015) is also present in psychotherapy. This is also in line with the early study by Dittes (1957) on the calming effect of the therapist’s friendliness and permissiveness. In “sharing the emotional load” through empathy, part of the emotional arousal from the original experience is as it were transferred to the empathizer. We also found an analogous trending effect in facial muscle activity in response to interventions; empathy increased the therapist’s and decreased the client’s facial muscle activation indexing negative emotional expressions. Also in facial expression, an empathizing therapist seems to take over some of the “load” from the patient. This highlights the dyadic system view by Beebe and Lachmann (2002) who suggest that the means through which emotion is regulated in interaction (in this case, facial expression) have systemic links to the internal regulation of the emotional arousal of the participants.

Given the lack of earlier research, we did not have specific hypotheses regarding the differences between the physiological responses in the two time frames: during whole sessions and during interventions. However, we made some intriguing findings regarding the time frame of the physiological correlates of challenge that call for further work, theoretical and empirical. While in terms of heart rate, both participants respond to challenge at the session level (heart rate increases), in terms of EDA a trending effect suggests that only clients respond to challenge at the session level. However, phasically, during and immediately after the very formulations, only the therapists’ arousal—both EDA and heart rate—increased, whereas clients did not show a phasic physiological response to challenge. This is different to what was found in the earlier studies on confrontation (McCarron and Appel, 1971; Olson and Claiborn, 1990), and may result from that the challenge that we measured was often very discreet. However, it appears that therapists respond to challenge predominantly phasically, whereas the clients respond predominantly tonically, at the session level. We suggest that this difference reflects the co-regulation of emotion in an inherently asymmetrical situation where the participants’ social roles are very different. At the local level, the therapists are sensitive to the emotional significance of the challenge they are performing, but they are not “carried away” by the challenge in global level of the session. This might be considered a form of professional regulation of emotion: being momentarily sensitive but still remaining neutral in the big picture (cf. Hochschild, 1979). Apart from being therapeutically motivated (i.e., beneficial for the psychotherapy process), such neutrality may enhance the therapists’ self-care (Norcross, 2000) in avoiding over-activation and work-related stress.

Furthermore, the results suggest that the “professional specificity” of the therapist’s emotion regulation pertains to challenge more than to empathy: in the therapists, the regulation of empathy, unlike challenge, seems to come closer to the regularities of generic human interaction—what above was depicted as the sharing of the emotional load. In other words, the therapist’s own emotion system seems to react more “profoundly” to being empathetic than to being challenging. On the other hand, the therapists’ arousal may be connected to the effort in reflective, “controlled” type of empathy that Messina et al. (2012) suggested in relation to the more lagged EDA synchrony in sessions with psychotherapists compared to sessions with psychologists. Finally, we would like to suggest that the temporal pattern of the clients’ response to challenge can index that the clients do not recognize the challenge immediately, but they process the challenging interventions during the session. Our study does not imply if this is conscious or not, but we suggest that such subtle phenomena can be taken under investigation in further studies combining close analysis of interactional dynamics and quantitative analysis of psychophysiology.

Another effect that we did not predict but that calls for further research is the interaction effect that empathy and challenge had in predicting heart rate and facial muscle activation. In sessions where the therapist shows much empathy and challenge, along with sessions with low empathy and low challenge, heart rate was higher and positive facial muscle activity (smile) stronger than in sessions where these variables were less balanced. On the other hand, heart rate decreased over the sessions where empathy and challenge were in balance. This suggests that the close proximity of empathy and challenge in therapeutic work found in earlier interaction research (Bänninger-Huber and Widmer, 1999; Voutilainen et al., 2010; Weiste and Peräkylä, 2014) is reflected also in physiological activation. Balance in empathy and challenge amounts to an intense but positive emotion, and on the other hand, decrease of arousal over the session, in both participants. To our interpretation, this reflects successful, rewarding, therapeutic collaboration. It also suggests that both “engagement” (where empathy and challenge are high) and “disengagement” (where empathy and challenge are low) can be beneficial, and needed in the course of the therapy. These aspects of interaction can be taken into consideration in future studies of therapy outcome.

This study has demonstrated the effort and sophistication in the psychotherapists’ professional work with their clients’ emotion. The study confirms that the therapists responses to their clients have immediate effects in both the therapist’s and the clients’ emotion system. Recognizing the physiological load and its successful regulation in standards sessions contributes to understanding of the psychotherapist’s professional role, and highlights the importance of the therapist’s self-care.

### Limitations

A limitation of the current study is the small amount of data and that some of the effects were found only as trending. Our physiological data shows that the client’s autonomic activation is constantly relatively high during the sessions, and this can amount to a roof-effect that prevents activation related to subtle differences from showing in the statistics. Data from more dyads would be helpful in solving this problem. Another limitation of this study considers the “fair to good” inter-coder reliability for challenge, and the “fair” inter-rater reliability for empathy. In our view, the common disagreement between coders and raters reflects the nature of the data (psychodynamic therapists’ discreet ways to express both empathy and challenge), and that the coding and rating schemes allowed context-sensitive interpretation. We acknowledge that more coders and raters would probably improve the reliability. Another methodological aspect in the coding is that our coding scheme is applicable especially in the type of psychodynamic therapy that our data represents. To study challenge in other types of therapies, a coding scheme sensitive to that context would be needed. Finding a best solution considering both good reliability and context-sensitivity in the coding of interactional phenomena remains as a challenge for further research.

## Author Contributions

AP, LV, NR, MS, and PH: conception and design of the study. LV, PH, MK, AP, and NR: acquisition, analysis, and interpretation of data. LV, PH, and AP: drafting the manuscript. AP, LV, NR, MS, PH, and MK: revising the manuscript, final approval of the version to be published, and agreement to be accountable for all aspects of the work in ensuring that questions related to the accuracy or integrity of any part of the work are appropriately investigated and resolved.

## Conflict of Interest Statement

The authors declare that the research was conducted in the absence of any commercial or financial relationships that could be construed as a potential conflict of interest.
